# Clinical trial design of serious gaming in mild cognitive impairment

**DOI:** 10.3389/fnagi.2015.00026

**Published:** 2015-03-11

**Authors:** Cristina Muscio, Pietro Tiraboschi, Ugo P. Guerra, Carlo A. Defanti, Giovanni B. Frisoni

**Affiliations:** ^1^Fondazione Europea Ricerca Biomedica, Centro Eccellenza AlzheimerOspedale Briolini, Bergamo, Italy; ^2^Department of Molecular and Translational Medicine, University of BresciaBrescia, Italy; ^3^Division of Neurology V/Neuropathology, Fondazione IRCCS Istituto Neurologico "Carlo Besta"Milan, Italy; ^4^Fondazione Poliambulanza, Nuclear Medicine DepartmentBrescia, Italy; ^5^Laboratory of Epidemiology, Neuroimaging and Telemedicine, IRCCS Centro San Giovanni di Dio-FatebenefratelliBrescia, Italy; ^6^Memory Clinic and LANVIE - Laboratory of Neuroimaging of Aging, University Hospitals and University of GenevaGeneva, Switzerland

**Keywords:** serious games, Alzheimer's disease, mild cognitive impairment, biomarker enrichment strategy, outcome measures, clinical trials

## Introduction

The “Global Impact of Dementia: 2013–2050” (Alzheimer's Disease International, 2013), released ahead of the December 2013 G8 Dementia Summit in London, estimated that 44.35 million people in the world were living with dementia in 2013. This number was predicted to increase to 75.6 million in 2030, and 135.5 million in 2050. This dramatic increase will have profound implications for social and economic costs (Alzheimer's Disease International, 2010). Since the most common dementia subtype (50–75%) is Alzheimer's disease (AD), its early detection and clinical effectiveness of its prevention and treatment represent a major public health concern and have been identified as a research priority (Alzheimer's Disease International, 2009; Ballard et al., 2011; Foster et al., 2014).

Recently, there has been a growing interest in employing Information and Communication Technologies (ICT) to evaluate patient's cognitive and functional impairment for early detection of AD (Wan Shamsuddin et al., 2011; Tarnanas et al., 2014). Beyond being important for assessment, ICT can also play a key role in the patient's treatment, stimulation, and rehabilitation (Robert et al., 2014). This is the idea underlying the current use of Serious Games (SGs), which are a broader reapplication of videogames resources integrating gaming and serious purposes. Lately, a few studies have started to investigate the efficacy of SGs used as an ICT intervention, which target cognitive decline, in people with AD and mild cognitive impairment (MCI). Until now, however, rigorous studies are still lacking. To overcome the current methodological issues and to evaluate the efficacy of SGs in secondary prevention (that currently is being pursued and is considered one of the potentially attainable goals of treatment, Foster et al., 2014), the purpose of the present opinion paper is to highlight the importance of defining harmonized SGs parameters, and to propose the implementation of biomarkers as enrichment strategy and outcome measures in SGs trial design. We will now review the history and state-of-art types and use of SGs, before describing in more detail our proposal.

## History of serious games: origin, typologies, target

SGs are games designed for a primary purpose other than entertainment, enjoyment or fun (Michael and Chen, 2005). The historical origin of this oxymoron dates back to Neo-Platonists, who referred the term “*serio ludere*” to light-hearted approach in literature dealing with serious matters (Manning, 2004). The first use of SG oxymoron close to its current use seems to be in book written (Abt, 1970), even though with a more extensive meaning. In fact, a SG could indeed be a computer game, a game, a role-playing game or even an outdoor game (Alvarez and Djaouti, 2012). The SG term in a digital context was firstly used in 2002, with the start of the Serious Game Initiative led by David Rejeski and Ben Sawyer in the US (De Gloria et al., 2014).

To date, SGs have been applied in many sectors, including education, training, defense, health, communications, marketing, politics, and the list is continually expanding (Alvarez and Djaouti, 2012). Since SGs addresses a set of markets, they are constituted by a wide variety of different types. Considering the dual nature of SGs, a system that classifies SGs according to both the “serious” and the “game” dimensions was proposed by Djaouti et al. (2011)^1^. This model has classified 3080 SGs so far.

Examples of SGs include (see Alvarez and Djaouti, 2012, for a review): (i) military games, commonly dedicated to tactical and strategic training as well as recruitment for the army; (ii) edugames for educational and training purposes, also usable in a school context; (iii) advergames, where the gameplay is centered around a commercial message; (iv) newsgames that are based on current events or certain journalistic issues; (v) SGs dedicated to health sector aimed to improve player's cognitive or physical abilities; etc.

SGs do not target exclusively young gamers. A considerable proportion (20–29%) of regular digital gamers are indeed older than 50 years (ESA, 2011; BIU, 2014). In this respect, it is worth noting the increase by 32% in the number of US females gamers aged 50 and older from 2012 to 2013 (ESA, 2014). Because the number of elderly people who play video games in the past decades has steadily increased and is predicted to grow further (Robert et al., 2014), even small beneficial effects may have significant public health implications (Alzheimer's Disease International, 2014).

## The state-of-art use of cognitive serious games with healthy older adults and AD patients

The cognitive effect of SGs played by older adults has not yet been studied thoroughly (Weybright et al., 2010; Alzheimer's Disease International, 2014). In the context of research focused on successful cognitive aging and on the possibility to modify the cognitive decline normally associated with healthy aging (Zinke et al., 2014), anyway, SGs have been demonstrated to be a motivating tool with some beneficial effects in improving cognitive functions in healthy older adults (Nouchi et al., 2012; Anguera et al., 2013). In the study reported by Anguera et al. (2013), indeed, it's worth mentioning that: (i) SG improved both trained and untrained cognitive abilities, which is commonly referred to as a transfer effect, that is the effect due to a training not only on skills or performance that are trained, but also on skills or performance that are not trained (Nouchi et al., 2012); (ii) untrained abilities that improved were sustained attention and working memory, which are known to be involved in everyday functioning; and (iii) performance gains remained stable 6 months after training without booster sessions. This transfer effect of SG on the improvement of executive functions and processing speeds in the elderly has been also demonstrated with a short-term training (Nouchi et al., 2012), suggesting that a possible transfer effect from laboratory-based tasks to real world ones may be expected. Neurophysiological findings support training-induced neuroplasticity as the mechanistic basis of these SG effects (Anguera et al., 2013).

However, whether AD patients or population at high risk for developing AD (i.e., MCI) may benefit from SGs is unknown (Robert et al., 2014).

Recently, some studies have started to employ SGs with people with AD and MCI as a cutting-edge cognition-focused intervention (Table 1). Cognition-focused interventions fall under the broader umbrella of non-pharmacological interventions, and can be defined as interventions that directly or indirectly target cognitive functioning as opposed to interventions that focus primarily on behavioral, emotional or physical functions (Bahar-Fuchs et al., 2013). These interventions are typically designed to promote intellectual stimulation and minimize cognitive impairment (Weybright et al., 2010). Progressive decline of cognitive functions is indeed a clinical feature of AD and has been found to be associated with impairment in activities of daily living (Tomaszewski Farias et al., 2009). Thus, intervention aimed at prevention and rehabilitation of such decline may promote a longer independent life at home and decrease the burden of dementia on patients and families.

**Table 1:** **Main findings of studies applying only cognitive or both physical and cognitive SGs to AD/MCI patients**.

**Author and year**	**Group**	**Sample size**	**Serious game**	**Study design**	**Outcome measure(s)**	**Main findings**
Stavros et al., 2010	MCI	59 (30 experimental, 29 control subjects)	Complete Brain Workout	N/A.	Neuropsychological change on cognitive tests.	Improvement of attention, verbal memory and ADL in the experimental group.
				Experimental program: tasks of visual attention, visual spatial abilities, visual memory, and executive function: 20 weekly sessions in 6 months.		
				Control program: no participation in any type of intervention.		
Weybright et al., 2010	MCI	2	Nintendo Wii™ Sport Bowling	Single-subject multiple baseline ABAB design.	Checklist based on observations of videotapes focusing on patients' attention and positive feelings.	Improvement of attention and positive feelings during Wii™ bowling game session.
				A = television-watching phase: 15-min, 4 times a week, 3–2 weeks.		
				B = Wii™ bowling game intervention: 15-min, 4 times a week, 2–3 weeks.		
Rosen et al., 2011	MCI	12 (6 experimental, 6 control subjects)	Adaptive games from Posit Science	Randomized pilot study.	Neuropsychological change on cognitive tests and brain function change as measured in an auditory-verbal fMRI task.	Improvement of memory and left hippocampal activation in the experimental group.
				Experimental program: 7 exercises designed to improve processing speed and accuracy in auditory processing: 100 min per session for 24 sessions.		
				Control program: listening to audio books, reading online newspapers, and playing a visuospatially-oriented computer game: 90 min per session for 24 sessions.		
Yamaguchi et al., 2011	AD, Parkinson's disease PD, Vascular dementia VD (mild-to-moderate)	7 AD, 1 PD, 1 VD	Xavix Hot Plus	Single group pre-/post-test design.	General cognitive, visuospatial and constructive functions, and behavioural changes on neuropsychological and multidimensional scales, respectively.	Post-test improvement of general cognitive, visuospatial and constructive functions, and sociability.
				Video sports-games specifically devised for rehabilitation (requiring, for example, to move legs to the sound of music or to grab coins which appearing on the TV screen) played once a week for 10 weeks.		
Boulay et al., 2011	AD (mild-to-moderately severe)	7	MinWii (MINDs)	Pilot usability study.	Efficacy (number of errors during each task), efficiency (time to complete each task, number of verbal interventions provided by the moderator) and satisfaction indicators (satisfaction questionnaire).	Clear learning effect and positive impact on satisfaction.
				1 training and 4 testing sessions focused on music therapy and cognitive stimulation, once per week for 3 months.		
Férnandez-Calvo et al., 2011	AD (mild)	45 (15 experimental, 30 control subjects)	Big Brain Academy	Randomized controlled trial.	Neuropsychological, behavioral, and functional changes on pertinent tests.	Slower rates of cognitive decline and fewer depressive symptoms in the experimental group.
				Experimental program: exercises designed to stimulate 5 cognitive domains such as thinking, memory, computation, reasoning ability, and identification: 3 times a week for 12 weeks.		
				Two control programs: (i) a traditional stimulation program 3 times a week for 12 weeks, or (ii) no participation in any type of intervention.		
Mosimann et al., 2014	MCI due to AD	158 (50 experimental, 53 active control, 55 passive control subjects)	Novel serious game	N/A.	SG performance, neuropsychological change on cognitive tests and default mode network connectivity change as measured by resting-state electroencephalography.	Greater spatial improvement in functional connectivity (associated with better performances in executive function, verbal encoding and retrieval tasks) and increased activation in prefrontal and temporo-parietal areas in the experimental group.
				Experimental program: a 5-week SG training protocol (a virtual museum cognitive stimulation environment), 4 times per week for 90 min each day.		
				Control programs not specified.		

Despite the promising results and the increasing interest in applying cognitive SGs to AD/MCI patients, rigorous feasibility and efficacy studies are still lacking, partly reflecting the only recent interest in employing SGs in cognitively impaired patients (Robert et al., 2014). The main methodological issues are: limitation of randomized controlled studies and lack of harmonized procedures (i.e., absence of standardized SG parameters such as when, where and with whom SGs have to be played), as well as small sample size and questionable choice of patient selection and outcome measures. However, these issues are common in studies addressing cognition-focused interventions (Woods et al., 2012; Bahar-Fuchs et al., 2013).

## Future perspectives

### Definition of harmonized SGs parameters

To overcome the current lack of harmonized procedures, one important aspect to be taken into account in the SG trial design includes the definition of parameters such as when, where and with whom SGs are more adapted to be played by AD/MCI patients.

According to the recommendations reported in (Robert et al., 2014), SGs for MCI patients' stimulation could be considered adapted to be used both everyday and once a week; at home, in day centers and in the nursing homes; with a therapist, a professional caregiver and a family caregiver.

In our opinion, SG trials should take into account these methodological recommendations, and assess SG feasibility and efficacy due to when, where, with whom SG is played by patients. As far as “with whom” is concerned, we think that it could also be interesting to study SGs played by multiple players physically co-present or by online patients connected from remote locations. One of SG strengths is indeed its possible role in promoting social interactions among patients with cognitive impairment (Robert et al., 2014).

### Biomarkers as enrichment strategy and outcome measures

It is generally estimated that up to one third of patients enrolled in AD trials do not have AD (Delrieu et al., 2014), leading to dilution of observable treatment effects (Aisen, 2011). However, AD pathology can be identified in living subjects through pathophysiological markers indicative of abnormal amyloid deposition that, in addition to a specific cognitive profile, moves a patient from a status of MCI of undetermined etiology to that of prodromal AD (Dubois et al., 2014). CSF Aβ1-42 and/or PET-amyloid imaging, as well as hippocampal atrophy on MRI, have indeed been qualified as enrichment biomarkers to enroll predemented AD subjects in regulatory clinical trials (see EMA/CHMP/SAWP/893622/2011 and EMA/CHMP/SAWP/809208/2011 qualification opinions). Inclusion of biomarkers into clinical trials for treatment of early AD has until now been recommended for pharmacological studies alone. However, both pharmacological and non-pharmacological studies can share the same issues that may have contributed to their failures (Doody et al., 2014; Salloway et al., 2014), including for example misdiagnosis of patients and insensitivity of outcome measures.

To overcome the above-mentioned limitations of cognition-focused interventions pertaining to patient selection and outcome measures, and to evaluate the efficacy of SG in secondary prevention, we propose to implement in the SG trial design: (i) a biomarker enrichment strategy to enroll MCI due to AD, and (ii) the use of biomarkers as outcome measures in combination with clinical ones.

A biomarker enrichment strategy would be expected to support screening out non-AD cases and screening in AD ones, reducing the diagnostic inaccuracy at enrollment and, thus, minimizing the masking of treatment effects that occurs when misdiagnosed patients are recruited (Morris and Selkoe, 2011). Once identified MCI due to AD using enrichment biomarkers, it could also be relevant to randomize these patients after stratifying them into different groups based on positivity on one or more biomarkers, in order to evaluate a possible differential effect of SG on MCI subjects presumably at different pre-dementia stages of the AD process. MCI patients with brain amyloidosis and neurodegeneration are indeed at higher risk of dementia in the following years. According to the current pathophysiologic model of AD (Jack et al., 2010), they might be at a more advanced disease stage (Prestia et al., 2013). For this reason, a SG effect dependent on single or multiple biomarker positivity could be hypothesized and taken into account for defining optimal SG protocols. Patients with MCI could also be stratify into two groups based on positivity or negativity of biomarkers, in order to investigate whether cognitively impaired subjects devoid of AD pathology might have a greater benefit due to less severe neuronal injury and, consequently, greater brain reserve (Stern, 2009). Finally, it could be interesting to verify if there is an association between biomarkers and methodological parameters cited in the previous subparagraph: for instance, can patients with a single positive biomarker or negativity of biomarkers take advantage from SGs played at lower time intensity (e.g., once at week) or alone at home compared with patients with multiple positive biomarkers or positivity of biomarkers, respectively?

Some studies in AD and MCI patients have shown that MRI and FDG-PET biomarkers may be more sensitive to change than clinical measures, as reported by Caroli et al. (2014) but regulatory agencies have not yet recognized biomarkers as surrogate outcome measures. This cautious approach is due to the requirement that, to be recognized as surrogate outcome measures, biomarker changes should reliably predict detectable clinical changes (Aisen, 2011). Unfortunately, the results of randomized clinical trials with anti-beta amyloid drugs (Abeta vaccine AN1792 and bapineuzumab) have until now shown no clinical efficacy despite a change in biomarkers. On the other hand, use of biomarkers would allow studies with fewer participants, shorter durations, lower costs, and with the possibility to control for the specificity of disease-modifying effect (Morris and Selkoe, 2011; Food and Drug Administration: Draft Guidance for Industry. Alzheimer's Disease: Developing Drugs for the Treatment of Early Stage Disease, 2013). Recent non-pharmacological studies that have incorporated hippocampal atrophy as biomarker outcome have found a disease-modifying benefit of aerobic exercise in early AD over 6 months (Honea et al., 2014), suggesting that similar results could also be found applying physical and cognitive SGs.

## Conclusions

If the presumed beneficial effects of SGs will be demonstrated by robust studies, the potential societal impact will be huge considering the very high prevalence of cognitive impairment due to AD, the popularity of video games played by baby-boomers now at risk of dementia, the current lack of effective treatments, and the cost-effectiveness of these enjoyable interventions. Moreover, video games already marketed to older adults for maintaining cognitive health may be seen as a scale up of a prevention program in a high-risk subgroup of the population (Alzheimer's Disease International, 2014). SGs may represent a motivating, low-barrier, engaging and sustainable method to improve or at least delay the decline in specific social, sensory-motor, cognitive and emotional functions of elderly people (Wiemeyer and Kliem, 2012).

### Conflict of interest statement

The authors declare that the research was conducted in the absence of any commercial or financial relationships that could be construed as a potential conflict of interest.

## Footnotes

^1^http://serious.gameclassification.com
